# Some Aspects of Medical Science

**Published:** 1894-06-09

**Authors:** 


### Some Aspects of Medical Science.

I talk of dreams,
Which are the children of an idle brain,
Begot of nothing but vain fantasy.

A particular interest has always surrounded the subject of dreams, intensified no doubt by the very mystery with which it is enshrouded. What are our dreams? has been the question of centuries—what is their nature, what their origin? And varied indeed are the answers in reply.

Mankind in early ages associated their dreams with superstitious beliefs, and possessed their character with something of the supernatural. Amongst Theistic nations they were regarded as revelations from God himself, and amongst Polytheists as inspired by some special deity; but Pagan and Theist alike had recourse to the wise man or soothsayer for their interpretation. And nowadays are we much nearer to knowing of what their nature is? Science has done much, and the romance which once environed has given place to conjectures regarding them which are fast assuming a more definite footing. Our attention has lately been called to this subject by Dr. Wilkes, whose exhaustive contribution in a much respected contemporary gives us much interesting data.

His solutions are based purely on scientific and physiological grounds—all that is mystic and conjectural on the subject being unconditionally dismissed. Although the old superstitions which linger to the present day around the World of Dreams, are not given any serious consideration at the hands of our authority, yet they are barely less powerful in their influence on our minds than in less civilized, less educated times. That the nature of dreams is phenomenal—mystic is still the unspoken tenet of many a cultivated person. It is a mystery which mankind has ever been ready to apostrophize.

And common-sense explanations are not always acceptable to us, if we are prejudiced in favour of other solutions. Sleep has always been spoken of as something inexplicable, above human understanding, its nature has not until quite lately been discussed in a scientific spirit. The fact of the tired body needing repose has not sufficiently accounted for this particular means to the end of regaining strength. The activity and repose of the brain is only, as Dr. Wilkes' paper points out, in accord with what is observed in the varying functions of all other organs.

These organs are constantly varying in their degree of activity, "and that activity," we quote the scientist's word, "has a close relation to the amount of blood circulating within it—the dictum *ubi stimulus fluxus* being generally accepted." This is more markedly true of the brain; when it is full of blood it is in a state of activity, and when anæmic it is in a state of repose. Mosso has proved this to demonstration. Another good example is to be found in the stomach; when empty it is a small organ, displaying no great vascularity, and producing little secretion; in this condition it is in repose, or asleep.

Consciousness implies activity, and unconsciousness repose or sleep. The process is a wonderful one, and has attracted, as has been already observed, the attention alike of philosopher, scientist, soothsayer, and poet. It is now generally admitted that thought does continue during sleep, and this is styled unconscious cerebration. We can most of us enumerate instances in our own experience, where the train of thought of our waking hours has been carried on through the night when we are sleeping. In some cases we have been afforded a solution, the sequence of our thoughts being often uninterrupted, and we wake up sometimes in the morning to find a vexed question settled, a problem solved. The brain thus is proved to remain unconscious, though consciousness itself is merely suspended.

The author of "The Nature of Dreams" illustrates how we can the better understand this theory of the brain being at work as in the waking state during the absence of consciousness itself. He explains that the mind being, as it were, cut off in sleep from the usual surroundings, it is unable to correct itself or its impressions, and so wanders on in an endless maze. As an illustration, he suggests, we are to imagine ourselves sitting over the fire on a winter's afternoon, falling into a deep fit of abstraction. We start up, look round, ascertain where we are, discard our mental vision, and are ourselves again. In very deep sleep, he reminds us, we cannot do this, and the vision we see becomes for a time a reality, and when we wake to consciousness we call it a dream. Doubtless they are quite incoherent, these dreams, and the dreamer is in the same mental condition as a lunatic.

Whatever opinion is held as to their origin, there seems but little doubt, Dr. Wilkes says, that the general assent is given to the fact that the result of the working of the brain or mind during sleep, all material surroundings being cut off, is that the dreamer is regarding the incidents, which are thoughts, as realities. It follows, therefore, that whatever he sees or hears in his dream is of his own creation, originating in himself; that when he argues a question with another person, it may be on politics or religion, he is dictating the speeches of both. Many illustrations can be given which further testify to this opinion.

Of course, this is looking at the subject from a common-sense point of view, and this is how it should be regarded according to the latest authority on the matter, who puts aside the supernatural element with which dreams are wont to be accredited in our minds. For amongst us there is a tendency still to consider this most natural and obvious phenomemon purely from a supernatural point of view.

A dream, in reality, is little else, when we rob it of our former illusions, but a picture formed in our minds during our sleep. What Dr. Wilkes is especially insistent upon is the fact that a dreamer can never produce from his brain what he has hitherto been *quite* ignorant of. His own conclusion, formed after laborious investigation, and based upon grave consideration, is that a dreamer merely forms a mental picture, and the description of it he calls a dream. Such a picture may be most rapidly formed—it is the recalling and the description which takes the time. This accounts for the apparent length of the dream in our memory when we wake up, a recalling which is out of proportion to the fact as it really is.

